# Efficacy and Safety of Qishen Yiqi Dripping Pill for Heart Failure With Preserved Ejection Fraction: A Systematic Review and Meta-Analysis

**DOI:** 10.3389/fphar.2020.626375

**Published:** 2021-02-09

**Authors:** Mengxi Wang, Yiwen Shan, Chenjie Wu, Peihua Cao, Weixin Sun, Jie Han, Le Shen, Jiandong Chen, Peng Yu, Xiaohu Chen

**Affiliations:** ^1^ Department of Cardiology, Affiliated Hospital of Nanjing University of Chinese Medicine, Nanjing, China; ^2^ Department of Cardiology, Jiangsu Province Hospital of Chinese Medicine, Nanjing, China; ^3^ First Clinical Medical College, Nanjing University of Chinese Medicine, Nanjing, China

**Keywords:** Qishen Yiqi dripping pill, heart failure with preserved ejection fraction, efficacy, safety, systematic review, meta-analysis

## Abstract

**Background:** The number of heart failure with preserved ejection fraction (HFpEF) patients is increasing year by year, yet all western medicines currently used for heart failure have been shown to be ineffective for HFpEF. Qishen Yiqi Dripping Pill is one of the commonly drugs for the treatment of heart failure in China. In recent years, some clinical studies found that it has curative effect on HFpEF.

**Objective:** To evaluate the efficacy and safety of Qishen Yiqi Dripping Pill in treatment of HFpEF.

**Methods:** Databases including CNKI, Wanfang, VIP, CBM, PubMed, Web of Science, The Cochrane Library and EMbase were searched from their inception to May 2020 to screen relevant randomized controlled trials. The “risk of bias” evaluation tool in the Cochrane Handbook was used to evaluate the quality of the included studies. RevMan 5.3 software was used for meta-analysis.

**Results:** Eight studies meeting the criteria were included, with a total of 895 patients. The results of meta-analysis showed that compared with western medicine alone, combination of western medicine and Qishen Yiqi Dripping Pill can further increase the quotient of early diastolic mitral inflow velocity and late diastolic mitral inflow velocity (E/A) in patients with HFpEF [mean difference (MD) = 0.20, 95% CI (0.14, 0.26), *p* < 0.000 01], decrease the quotient of early diastolic mitral inflow velocity and mitral annular tissue velocity (E/e′) [MD = −2.50, 95% CI (−3.18, −1.82), *p* < 0.000 01], decrease brain natriuretic peptide (BNP) [MD = −151.83, 95% CI (−245.78, −57.89), *p* = 0.002], increase cardiac function improvement rate [relative risk (RR) = 1.30, 95% CI (1.11, 1.52), *p* = 0.001], and increase six-minutes walking distance (6-MWD) [MD = 64.75, 95% CI (22.65, 106.85), *p* = 0.003]. Four studies reported the occurrence of adverse reactions, among which three studies reported no adverse reactions and one study reported three patients with mild adverse reactions in the intervention group.

**Conclusion:** Current evidence suggests that Qishen Yiqi Dripping Pill may be effective in the treatment of HFpEF. However, due to the low quality of the included studies, lack of placebo control, large heterogeneity among different studies, and great possibility of publication bias, the results of our review should be evaluated with more prudence, more high-quality clinical studies are needed to verify the conclusion in the future. In addition, the safety of Qishen Yiqi Dripping Pill remains uncertain, further assessment is required in the future.

## Introduction

Heart failure (HF) is a clinical syndrome of cardiac structural or functional abnormalities which is the final stage of many cardiovascular diseases. The prevention and treatment of HF have achieved significant progress. However, with the developing of medical level, the survival time of the patients with cardiovascular diseases has been prolonged, resulting in the consistently increased incidence of HF (Ponikowski et al., 2016). Formerly, scholars believed that decreased left ventricular ejection fraction (LVEF) is one of the essential requirements for the diagnosis of HF. As the research going, it was found that more than half of the patients with HF were not accompanied by a reduction in LVEF, and the amount of such patients increased year by year (Steinberg et al., 2012). HF was divided into HF with preserves ejection fraction (HFpEF, LVEF > 50%), HF with midrange ejection fraction (HFmrEF, LVEF 40–49%) and HF with reduced ejection fraction (HFrEF, LVEF < 40%) by the European Society of Cardiology (ESC) in 2016 (Ponikowski et al., 2016). Different from the patients with HFrEF, the patients with HFpEF lack specificity in clinical manifestations and auxiliary examinations. Therefore, its clinical recognition rate is low and there is still lack of uniform diagnostic criteria. Statistics suggest that about 1/6 HF patients can not be recognized among the elderly people with exertional dyspnea and most of them are HFpEF patients (Ponikowski et al., 2016). At the same time, patients with HFpEF have poor quality of life, high rehospitalization rates and mortality. Hence, this disease has been a huge threat to human health and brought tremendous burden to our society (Zile et al., 2004; Huang et al., 2017).

In the past, the treatment for HF mainly focused on enhancing myocardial contraction and alleviating symptoms. With the deepening of research, the aim of treatment has turned to how to improve the long-term prognosis of patients. ACEI/ARB (Konstam et al., 2009; Guo and Li, 2016), β-adrenergic receptor blockers (Packer et al., 2001), aldosterone receptor antagonists (Hernandez et al., 2012), ARNI (McMurray et al., 2014) and SGLT-2 (McMurray et al., 2019) have already been proved to enhance the quality of life, as well as reduce hospitalization rates and mortality of HFrEF patients. Unfortunately, the above medicines failed to improve the prognosis of patient with HFpEF (Cleland et al., 2006; Massie et al., 2008; Conraads et al., 2012; Edelmann et al., 2013; Solomon et al., 2019), which means the previous treatment regiments of HFrEF have not been satisfactory for HFpEF patients. Exploring the pathophysiological mechanism and individualized treatment measure will be the main research directions of HFpEF in the future.

Traditional Chinese medicine (TCM) has accumulated a lot experience in the treatment of HF. According to TCM theory, deficiency of yangqi as well as blood stasis (yuxue) and retained fluid (shuiyin) are the main pathogenesis of HF. The treatment principle is to supplement qi and warm yang, activate blood and promote diuresis. For the past few years, some scholars putted forward that the pathogenesis of HFpEF is not exactly the same as HFrEF. Qi deficiency and yuxue were considered as the major pathogenesis of HFpEF. Correspondingly, the treatment principle should be supplementing qi and activating blood (Liu and Xu, 2017). Qishen Yiqi Dripping Pill is a Chinese patent medicine with the function of yiqi and huoxue approved by the National Medical Products Administration of China. It is composed of *Astragalus propinquus* Schischkin (Huang qi), Salvia miltiorrhiza Bunge (Dan shen), Panax pseudo-ginseng (San qi) and Dalbergia odorifera (Jiang xiang). Numerous clinical studies have demonstrated its effectiveness in the treatment of HF (Wang et al., 2013) and has been recommended for the treatment of HF by “The Consensus of Chinese Experts of Combined TCM and Western Medicine” (Chen et al., 2016). In recent years, some researchers have attempted to conduct clinical studies on the treatment of HFpEF with Qishen Yiqi Dripping Pill and its effectiveness has been preliminarily verified. However, the sample size of these studies is too small to provide convincing evidence. Therefore, we conducted this systematic review and meta-analysis to evaluate the efficacy and safety of Qishen Yiqi Dripping Pill in the treatment of HFpEF aiming to provide more evidence for TCM treatment on this disease.

## Materials and Methods

This systematic review and meta-analysis followed the Preferred Reporting Items for Systematic Reviews and Meta-Analyses (PRISMA) statement and has been registered in PROSPERO (registration number: CRD42020193864).

### Database and Search Strategies

We searched the following databases from their inception to May 2020: China National Knowledge Infrastructure (CNKI), Chinese Scientific Journals Database (VIP), Wan-fang Database (wanfang), Chinese Biomedicine Database (CBM), PubMed, Embase, Web of Science, and The Cochrane Library. The retrieval language was restricted to Chinese and English. The searched words including: “Qishen Yiqi”, “Qishen Yiqi dripping pill”, “Qishen Yiqi Diwan”, “heart failure with preserved ejection fraction”, “heart failure with normal ejection fraction”, “ejection fraction preserves heart failure”, “diastolic heart failure”, “diastolic dysfunction”, “preserved cardiac function heart failure”, and “HFpEF”. The search strategy of combining free words with subject words was adopted. We also manually retrieve the references of published reviews to search additional relevant studies.

### Inclusion Criteria

Studies meeting the following criteria were included: 1) randomized controlled trials (RCTs); 2) patients were diagnosed with HFpEF based on the ESC, American Heart Association (AHA) or Chinese Society of Cardiology guidelines for HF (Ponikowski et al., 2016; Yancy et al., 2017; Cardiovascular Society Heart failure Group of Chinese Medical Association et al., 2018); 3) the control group received conventional treatment including diuretic, aldosterone receptor antagonist, β-blocker, ACEI, ARB, ivabradine, and drugs that improve myocardial metabolism; 4) the intervention group received conventional treatment combined with Qishen Yiqi Dripping Pill (produced by Tianjin Tianshili Pharmaceutical Co., Ltd., oral administration, 0.5 g at a time, three times a day); 5) the primary outcomes include as follows, 1) the quotient of early diastolic mitral inflow velocity and late diastolic mitral inflow velocity (E/A), 2) the quotient of early diastolic mitral inflow velocity and mitral annular tissue velocity (E/e′); (6) the secondary outcomes include as follows, 1) brain natriuretic peptide (BNP), 2) cardiac function improvement rate (according to “Guiding Principles for Clinical Research of New Chinese Medicines”) (Ministry of health of the people’s republic of China, 2002), 3) six-minutes walking distance (6-MWD).

### Exclusion Criteria

Studies meeting the following criteria were excluded: 1) the data is incomplete; 2) case reports, reviews, conference literature, theoretical discussions and experience summaries; 3) the baseline information of patients were inconsistent.

### Data Extraction

Two researchers independently screened the retrieved literature according to the inclusion and exclusion criteria. They extracted the data and checked with each other, discussed and solved divergences with the third researcher. Data were extracted including first author name, year of publication, number of patients, gender, average age, intervention measures, treatment duration, outcomes and adverse reactions.

### Quality Evaluation

The methodological quality of included studies was assessed according to the “risk of bias” evaluation tool in the Cochrane Handbook. Seven elements will be evaluated: 1) random sequence generation; 2) allocation concealment; 3) blinding of researchers and subjects; 4) blinding of outcome assessors; 5) completeness of outcome data; 6) selective reporting; 7) other bias. All the above were evaluated as “low risk”, “high risk”, or “unclear risk”.

### Data Analysis

RevMan 5.3 software was used to perform the statistical analysis. Relative risk (RR) was used for dichotomous variables, mean difference (MD) was used for continuous variables and 95% confidence interval (CI) was calculated for both variables. Heterogeneity will be assessed by the *χ* ^2^ test and the I^2^ statistic. If substantial heterogeneity existed (*I* ^2^ > 50% or *p* < 0.05), a random effect model was applied; otherwise, a fixed effect model was applied. We also conducted subgroup analysis and sensitivity analysis to explore the sources of heterogeneity and verify the stability of the meta-analysis results. Funnel plots, Egger’s test and Begg’s test were chosen to assess publication bias.

## Results

### Search Results

**FIGURE 1:**
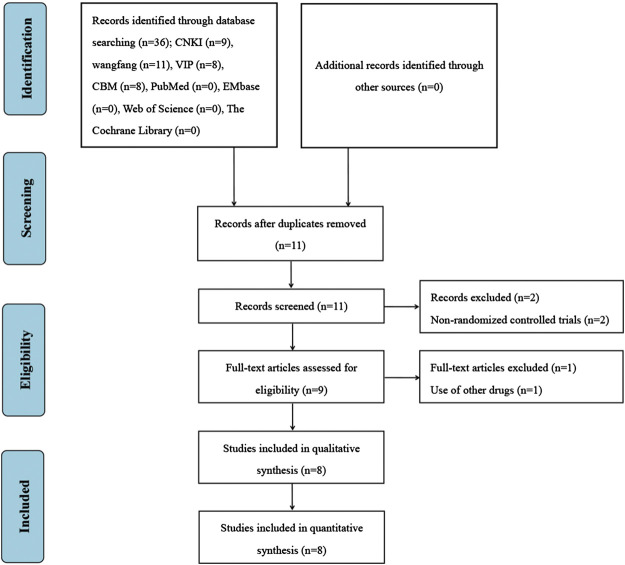
Flow diagram of literature screening.

A total of 36 studies were retrieved from the above database. Endnote software was used to eliminate 25 duplicate studies. After reading the titles and abstracts, two studies were excluded because they were non-randomized controlled trials. After reading the full texts, one study was excluded because its intervention group used other drugs. Finally, eight studies were included for the systematic review and meta-analysis (Zhang and Li, 2013; Li, 2014; He, 2015; Hu et al., 2015; Qiu, 2016; He and Yang, 2019; Zhang K. X. et al., 2019; Song, 2020). The screening process is presented in Figure 1.

### Study Characteristics

The eight studies involved a total of 895 patients (452 in intervention group and 443 in control group). The sample size ranged from 80 to 169. The average age ranged from 56.20 to 70.53. The control group received conventional treatment including diuretic, aldosterone receptor antagonist, β-blocker, ACEI and ARB. The intervention group received conventional treatment combined with Qishen Yiqi Dripping Pill (oral administration, 0.5 g at a time, three times a day). The treatment duration ranged from 56 to 180 days. Eight studies (Zhang and Li, 2013; Li, 2014; He, 2015; Hu et al., 2015; Qiu, 2016; He and Yang, 2019; Zhang K. X. et al., 2019; Song, 2020) reported the E/A as outcome, three studies (Li, 2014; Qiu, 2016; Song, 2020) reported the E/e′ as outcome, five studies (Zhang and Li, 2013; Li, 2014; He, 2015; Hu et al., 2015; Qiu, 2016) reported the BNP as outcome, 5 studies (Zhang and Li, 2013; Li, 2014; He, 2015; Hu et al., 2015; Qiu, 2016) reported the cardiac function improvement rate as outcome and 4 studies (Zhang and Li, 2013; Li, 2014; Qiu, 2016; Zhang K. X. et al., 2019) reported the 6-MWD as outcome. Only four included studies reported adverse events (Zhang and Li, 2013; He, 2015; Hu et al., 2015; Zhang K. X. et al., 2019), three of them (Zhang and Li, 2013; He, 2015; Hu et al., 2015) found no adverse events occurred during follow-up and one (Zhang K. X. et al., 2019) reported that three patients in the intervention group developed adverse events. The detailed characteristics of the eight studies were presented in Table 1.

**TABLE 1:** Basic characteristics of included studies.

Study	Sample size	Age (years)	Sex M/F	Intervention measures		Treatment duration (days)	Outcomes	Adverse events
	I/C	I/C	I/C	I	C			
He (2015)	40/40	56.20	48/32	D+ACEI or ARB+ARA+β+QSYQ 0.5 g tid	D+ACEI or ARB+ARA+β	180	①③④	None
He and Yang (2019)	60/60	I: 61.40 ± 6.21	I: 38/22	D+ACEI or ARB+β+QSYQ 0.5 g tid	D+ACEI or ARB+β	84	①	Not reported
		C: 62.21 ± 7.13	C: 36/24					
Hu et al. (2015)	40/40	I: 67.10 ± 5.20	I: 15/25	D+ACEI or ARB+β+QSYQ 0.5 g tid	D+ACEI or ARB+β	56	①③④	None
		C: 66.20 ± 6.10	C: 17/23					
Li (2014)	40/40	62.30 ± 8.60	—	D+ACEI or ARB+β+QSYQ 0.5 g tid	D+ACEI or ARB+β	90	①②③④⑤	Not reported
Qiu, (2016)	80/80	63.40 ± 9.70	—	D+ACEI or ARB+β+QSYQ 0.5 g tid	D+ACEI or ARB+β	90	①②③④⑤	Not reported
Song (2020)	55/55	I: 63.60 ± 9.20	I: 31/24	D+ACEI or ARB+β+QSYQ 0.5 g tid	D+ACEI or ARB+β	56	①②	Not reported
		C: 64.80 ± 8.90	C: 30/25					
Zhang and Li (2013)	49/47	I: 67.00 ± 6.00	—	D+ACEI or ARB+β+QSYQ 0.5 g tid	D+ACEI or ARB+β	180	①③④⑤	None
		C: 68.00 ± 8.00						
Zhang K. X. et al. (2019)	88/81	I: 70.53 ± 5.28	—	ACEI or ARB+ARA+β+QSYQ 0.5 g tid	ACEI or ARB+ARA+β	180	①⑤	Nausea
		C: 70.12 ± 6.98						

I, intervention group; C, control group; M, male; F, female; D, diuretic; ARA, aldosterone receptor antagonist; β, β-blocker; QSYQ, Qishen Yiqi Dripping Pill; ① E/A; ② E/e′; ③ BNP; ④ cardiac function improvement rate; ⑤ 6-MWD.

### Quality Assessment of Included Studies

**FIGURE 2:**
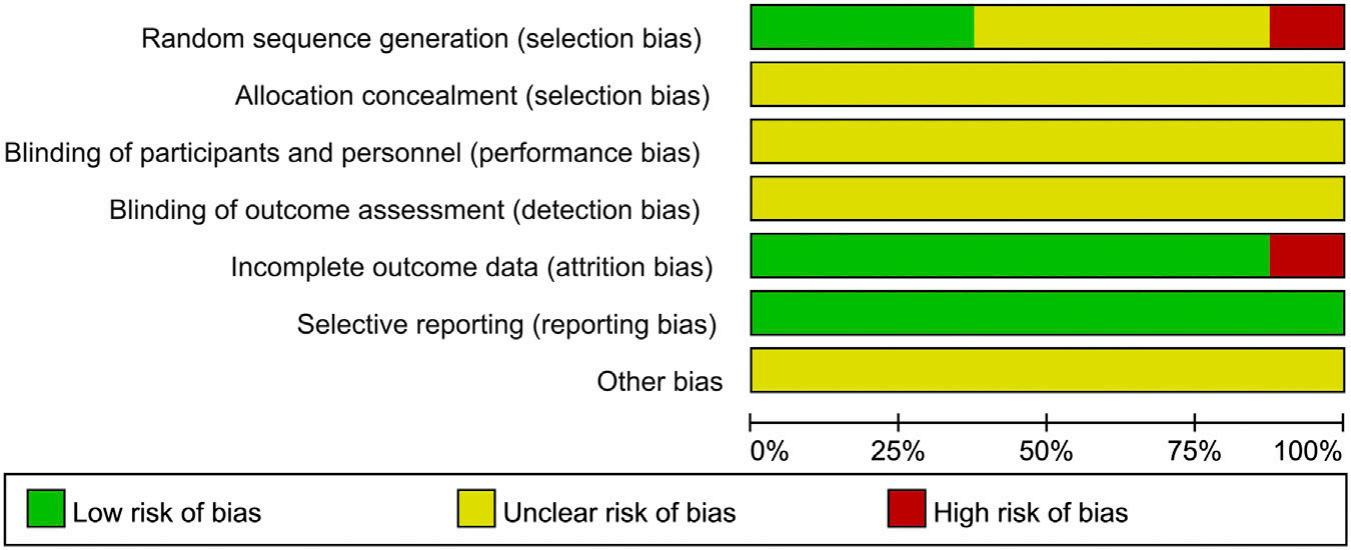
Risk of bias graph.

**FIGURE 3:**
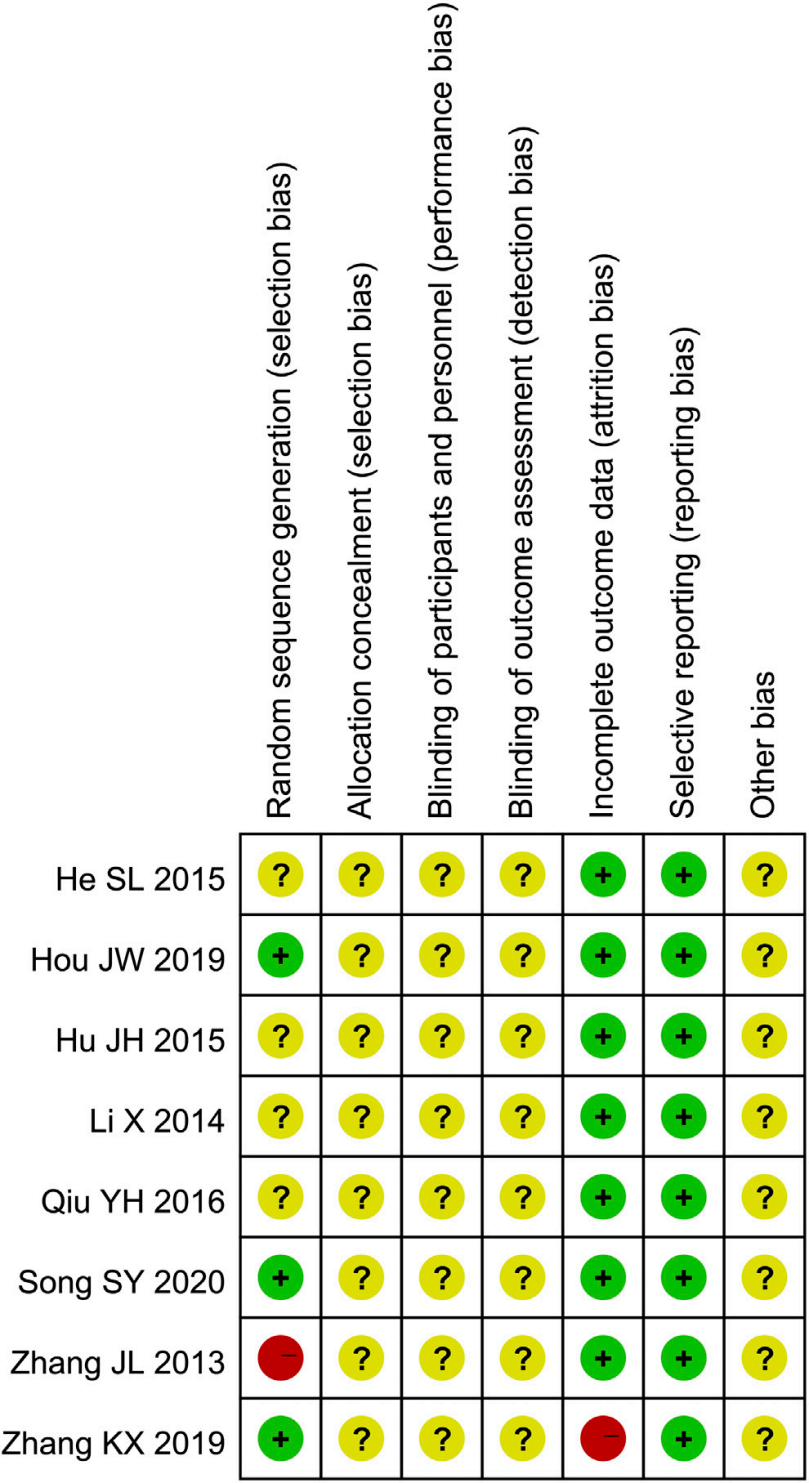
Risk of bias summary.

Only four studies (Zhang and Li, 2013; He and Yang, 2019; Zhang K. X. et al., 2019; Song, 2020) described the randomization method. Three of them (He and Yang, 2019; Zhang K. X. et al., 2019; Song, 2020) used appropriate randomization method (random number table method) and one of them (Zhang and Li, 2013) used inappropriate randomization method (according to the time of admission). None of the eight studies described allocation concealment procedure and blinding. Two studies (Zhang and Li, 2013; Zhang K. X. et al., 2019) reported 15 patients lost to follow-up. All of the 8 studies reported predetermined outcomes, and none reported other biases. The results of quality assessment are presented in Figures 2 and 3.

### Efficacy Assessment

#### E/A

**FIGURE 4:**
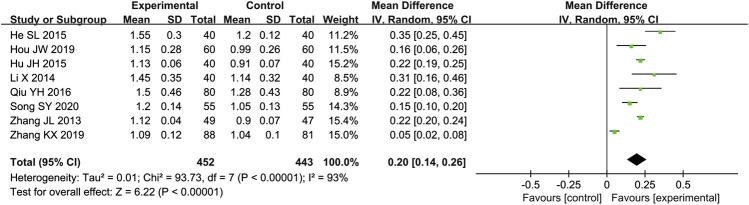
Forest plot of E/A.

Eight studies (Zhang and Li, 2013; Li, 2014; He, 2015; Hu et al., 2015; Qiu, 2016; He and Yang, 2019; Zhang K. X. et al., 2019; Song, 2020) reported the E/A as outcome. Due to the high heterogeneity (*p* < 0.000 01, I^2^ = 93%), we used a random effect model to analyze the data. The meta-analysis results indicated that combination of western medicine and Qishen Yiqi Dripping Pill can further increase E/A compared with western medicine alone [MD = 0.20, 95% CI (0.14, 0.26), *p* < 0.000 01] (Figure 4).

#### E/e′

**FIGURE 5:**
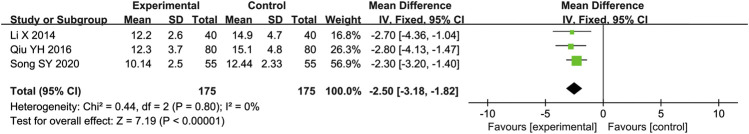
Forest plot of E/e′.

Three studies (Li, 2014; Qiu, 2016; Song, 2020) reported the E/e′ as outcome. Due to the low heterogeneity (*p* = 0.80, I^2^ = 0%), we used a fixed effect model to analyze the data. The meta-analysis results indicated that combination of western medicine and Qishen Yiqi Dripping Pill can further decrease E/e′ compared with western medicine alone [MD = −2.50, 95% CI (−3.18, −1.82), *p* < 0.000 01] (Figure 5).

#### BNP

**FIGURE 6:**
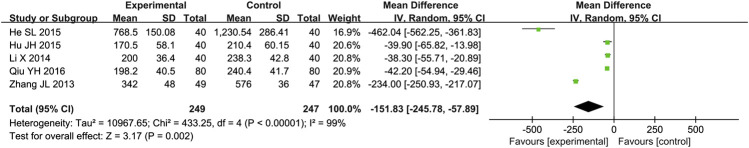
Forest plot of BNP.

Five studies (Zhang and Li, 2013; Li, 2014; He, 2015; Hu et al., 2015; Qiu, 2016) reported the BNP as outcome. Due to the high heterogeneity (*p* < 0.000 01, I^2^ = 99%), we used a random effect model to analyze the data. The meta-analysis results indicated that combination of western medicine and Qishen Yiqi Dripping Pill can further decrease BNP compared with western medicine alone [MD = −151.83, 95% CI (−245.78, −57.89), *p* = 0.002] (Figure 6).

#### Cardiac Function Improvement Rate

**FIGURE 7:**
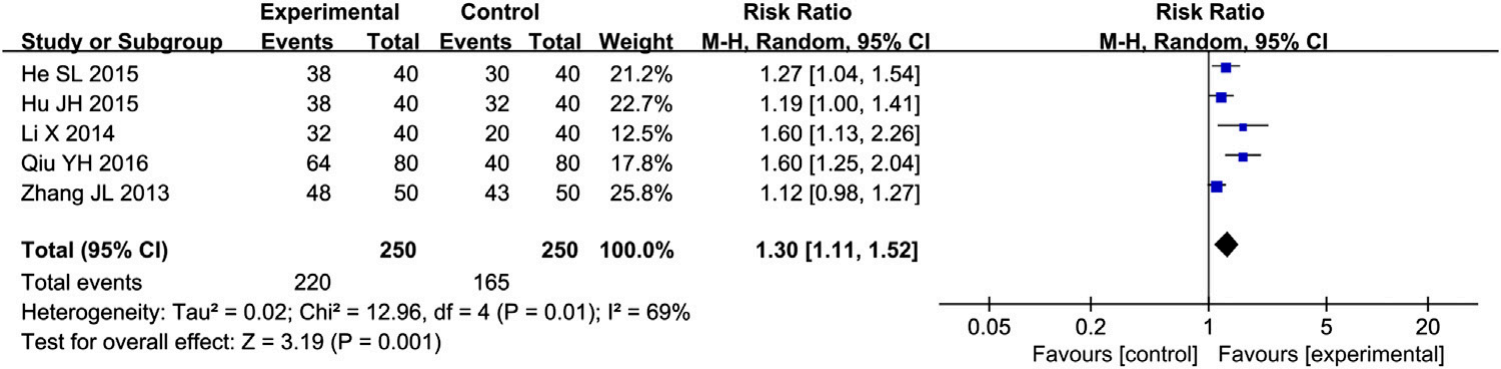
Forest plot of cardiac function improvement rate.

Five studies (Zhang and Li, 2013; Li, 2014; He, 2015; Hu et al., 2015; Qiu, 2016) reported the cardiac function improvement rate as outcome. Due to the high heterogeneity (*p* = 0.01, *I* ^2^ = 69%), we used a random effect model to analyze the data. The meta-analysis results indicated that combination of western medicine and Qishen Yiqi Dripping Pill can further increase cardiac function improvement rate compared with western medicine alone [RR = 1.30, 95% CI (1.11, 1.52), *p* = 0.001] (Figure 7).

#### 6-MWD

**FIGURE 8:**
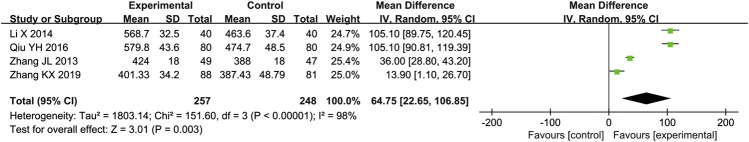
Forest plot of 6-MWD.

Four studies (Zhang and Li, 2013; Li, 2014; Qiu, 2016; Zhang K. X. et al., 2019) reported the 6-MWD as outcome. Due to the high heterogeneity (*p* < 0.000 01, *I* ^2^ = 98%), we used a random effect model to analyze the data. The meta-analysis results indicated that combination of western medicine and Qishen Yiqi Dripping Pill can further increase 6-MWD compared with western medicine alone [MD = 64.75, 95% CI (22.65, 106.85), *p* = 0.003] (Figure 8).

### Safety

Only four included studies reported adverse events (Zhang and Li, 2013; He, 2015; Hu et al., 2015; Zhang K. X. et al., 2019). Three of them (Zhang and Li, 2013; He, 2015; Hu et al., 2015) found no adverse events occurred during follow-up and one (Zhang K. X. et al., 2019) reported that three patients in the intervention group developed mild nausea with spontaneous remission. The above results seemed to indicate that the safety of Qishen Yiqi Dripping Pill is good. However, due to the small sample size, more studies are needed to further confirm the safety in the future.

### Subgroup Analysis

To explore the sources of heterogeneity, we conducted a subgroup analysis of E/A, BNP, cardiac function improvement rate and 6-MWD, based on treatment duration (more than 4 months or less than 4 months) and average age (more than 65 years old or less than 65 years old). The effect of combination of western medicine and Qishen Yiqi Dripping Pill on these four outcomes was consistent with the results described above in most subgroup. Only in the subgroup analysis of BNP, the negative conclusion was obtained in the subgroup with average age greater than 65 years. However, there is still a downward trend in BNP. We consider that this may be related to the small sample size of this subgroup. If the sample size increases, the conclusion may be reversed. Moreover, the heterogeneity of E/A, BNP and 6-MWD was significantly reduced when subgroup analysis was conducted based on treatment duration. The heterogeneity of cardiac function improvement rate was significantly reduced when subgroup analysis was conducted based on average age. These suggest that treatment duration may be one of the sources of heterogeneity on E/A, BNP and 6-MWD. Meanwhile, average age may be one of the sources of heterogeneity on cardiac function improvement rate. The results of subgroup analysis are presented in Tables 2 and 3 and Supplementary Figures S1–S8.

**TABLE 2:** Subgroup analysis based on treatment duration of E/A, BNP, cardiac function improvement rate and 6-MWD.

Outcome	Treatment duration	n	MD/RR (95%CI)	I^2^ (%)	Z	*p*
E/A	more than 4 months	3	0.20 (0.06, 0.34)	98	2.76	0.006
	less than 4 months	5	0.20 (0.15, 0.24)	53	8.30	<0.00001
BNP	more than 4 months	2	−342.45 (−565.66, −119.24)	95	3.01	0.003
	less than 4 months	3	−40.71 (−50.27, −31.16)	0	8.35	<0.00001
CFIR	more than 4 months	2	1.17 (1.03, 1.32)	20	2.46	0.01
	less than 4 months	3	1.42 (1.10, 1.83)	69	2.70	0.007
6-MWD	more than 4 months	2	25.61 (3.99, 47.23)	88	2.32	0.02
	less than 4 months	2	105.10 (94.64, 115.56)	0	19.69	<0.00001

CFIR, cardiac function improvement rate.

**TABLE 3:** Subgroup analysis based on average age of E/A, BNP, cardiac function improvement rate and 6-MWD.

Outcome	Treatment duration	n	MD/RR (95%CI)	I^2^ (%)	Z	*p*
E/A	more than 65 years old	3	0.16 (0.06, 0.26)	97	3.19	0.001
	less than 65 years old	5	0.23 (0.14, 0.32)	74	5.26	<0.00001
BNP	more than 65 years old	2	−137.21 (−327.43, 53.01)	99	1.41	0.16
	less than 65 years old	3	−137.98 (−213.66, −62.31)	97	3.57	0.0004
CFIR	more than 65 years old	2	1.15 (1.03, 1.27)	0	2.62	0.009
	less than 65 years old	3	1.49 (1.28, 1.73)	38	5.19	<0.00001
6-MWD	more than 65 years old	2	25.61 (3.99, 47.23)	88	2.32	0.02
	less than 65 years old	2	105.10 (94.64, 115.56)	0	19.69	<0.00001

CFIR, cardiac function improvement rate.

### Sensitivity Analysis

To verify the stability of meta-analysis results, we conducted a sensitivity analysis for all outcomes by removing the studies one by one, re-performing meta-analysis of the remaining studies and observing whether the results changed significantly. We found that there are no significant changes in heterogeneity and effect size, which indicates that the meta-analysis results are stable.

### Publication Bias

We didn’t perform a publication bias assessment because the included studies are less than 10. However, it should be noted that since all included studies were conducted in China and the results were all positive, there is a great possibility of publication bias.

## Discussion

Through the systematic review of 895 patients, we found that compared with western alone, combination of Qishen Yiqi Dripping Pill and western medicine could further decrease E/e′ and BNP, increase E/A, cardiac function improvement rate and 6-MWD. In terms of safety, our review results shown that only three patients with mild nausea and no serious adverse events was found. It seems that Qishen Yiqi Dripping Pill is safe. However, it should be noted that only four studies with 217 sample size in total reported adverse events, which was too small to provide strong evidence. Some other clinical studies which are not included found that Qishen Yiqi Dripping Pill had adverse reactions such as decreased blood pressure and heart rate, abnormal liver and kidney function during use (Zhang Y. L. et al., 2019), which did not exist in our included studies. In addition, the description of adverse reactions in the instructions for Qishen Yiqi Dripping Pill is “unclear”, indicating that there is no reliable clinical evidence to support the drug’s safety. Therefore, we consider that the safety of Qishen Yiqi Dripping Pill in the treatment of HFpEF remains uncertain, more clinical studies are needed to confirm it in the future.

It is well known that in small-scale clinical studies, surrogate end-point is often chosen rather than clinical end-point, because the former requires less sample size and observation period than the latter. Especially for some preliminary studies, directly performing large sample and long-term researches to observe clinical end-point may consume resources unnecessarily in the case that lack of enough evidence, and it is often difficult to get positive results. Therefore, we found that all the studies included in this systematic review chose surrogate end-point. It still have certain clinical value, even could not provide direct evidence of prognosis.

The mechanism of HFpEF is closely related to cardiac diastolic dysfunction (Paulus et al., 2007). E/e′ and E/A are the common indicators in evaluating cardiac diastolic function. ESC consensus on the diagnosis of HFpEF has taken E/e′ ≥ 15 as one of the main diagnostic criteria for HFpEF in 2019 (Pieske et al., 2019). Some scholars found that simultaneous detection of E/e′ and E/A can help improve the accuracy of diagnosis and treatment of HFpEF (Mitter et al., 2017). In addition, E/e′ and E/A have been verified to be associated with hospitalization rates, cardiovascular mortality, and all-cause mortality in HFpEF patients (Halley et al., 2011). Therefore, we selected E/e′ and E/A as the main outcomes. Previous studies indicated that western medicine have no significant effect on E/e′ and E/A in HFpEF patients (Xiang et al., 2019). The results of our review showed that Qishen Yiqi Dripping Pill could significantly decrease E/e′, increase E/A, and similar conclusions were obtained in subgroups of different treatment duration and age. This suggests that Qishen Yiqi Dripping Pill may have certain advantages in improving the cardiac diastolic function of HFpEF patients. Moreover, due to the relevance between the prognosis and the two outcomes (E/e′ and E/A), our research results suggest that Qishen Yiqi Dripping Pill may improve the prognosis by decreasing the E/e′ and increasing the E/A of HFpEF patients. However, great heterogeneity in the analysis of E/A should be noticed. Although we successfully reduced heterogeneity in the two subgroups (treatment duration less than 4 months and age less than 65 years) by conducting subgroup analysis based on treatment duration and average age, heterogeneity was still high in other subgroups (treatment duration more than 4 months and age more than 65 years). We considered heterogeneity may come from the impact that various factors such as measuring instruments, methods and personnel have on the E/A. Therefore, we should take a cautious attitude toward the analysis results of this outcome.

BNP, as the most commonly serum marker in the diagnosis and treatment of HF, mainly reflects the degree of ventricular load. A large number of studies have confirmed its value in the diagnosis, differential diagnosis, severity assessment and prognosis assessment of HF (Booth et al., 2014; Roberts et al., 2015). In recent years, with the deepening understanding of the classification of HF, scholars began to turn to the research on the correlation between BNP and different types of HF, and preliminarily proved that BNP is also valuable for guiding the diagnosis and evaluating the prognosis of HFpEF. In 2019, ESC consensus on the diagnosis of HFpEF also took BNP > 80 pg/ml (sinus rhythm) or BNP > 240 pg/ml (atrial fibrillation) as one of the main diagnostic criteria (Paulus et al., 2007). Hamatani's study showed that BNP level before discharge was correlated with hospitalization rate and all-cause mortality of HFpEF patients (Hamatani et al., 2018). The results of our review found that combination of Qishen Yiqi Dripping Pill and western medicine can further decrease the BNP level in patients with HFpEF. However, it should be noticed that we obtained a negative result in subgroup with average age greater than 65 years [MD = −137.21, 95% CI (−327.43, 53.01), *p* = 0.16]. Nevertheless, BNP was found in a downward trend. Therefore, we think that this result may be related to the small sample size, if the sample size is enlarged, the conclusion might be reversed. Similar to the analysis result of E/A, we also attempted to conduct subgroup analysis to explore the sources of heterogeneity, but heterogeneity was reduced only in subgroup with treatment duration less than 4 months. Since the BNP could be affected by various factors such as age, anemia, infection, renal function and so on, we consider that heterogeneity may derive from these.

Although long-term prognosis is the key to evaluate the effectiveness of drugs, the degree of improvement in clinical symptoms cannot be ignored. Particularly for HFpEF, it is important to alleviate the symptoms and improve the living quality of patients before there is effective way to improve the long-term prognosis. Cardiac function improvement rate and 6-MWD are the most common indicators to evaluate the degree of improvement in clinical symptoms of HF. The results of our review showed that combination of Qishen Yiqi Dripping Pill and western medicine can further increase the cardiac function improvement rate and 6-MWD in HFpEF patients. Meanwhile, similar results were obtained in subgroups of different treatment duration and age. This indicates that Qishen Yiqi Dripping Pill could alleviate the clinical symptoms, improve the grade of cardiac function and living quality of HFpEF patients. We conducted a subgroup analysis of the cardiac function improvement rate based on average age and found that the heterogeneity was significantly reduced in both subgroups with average age greater than 65 years and less than 65 years. This suggests that age may be the main source of heterogeneity. In the subgroup analysis of 6-MWD, we obtained results similar to those of E/A, i.e., there was still considerable heterogeneity in the subgroups of treatment duration more than 4 months and age more than 65 years. We consider that this might be due to the great influence of evaluator subjective factors and measurement errors on 6-MWD.

The pathogenesis of HFpEF is still unclear. Current studies suggest that increased sympathetic excitability can cause cardiac diastolic dysfunction, increase myocardial stiffness by increasing mechanical load of the heart, and activate cardiomyocyte inflammatory response (Rosendorff, 2009). On the one hand, activation of inflammatory response could upregulates the expression of transformed growth factor, promotes myofibroblast production, and thus increases collagen content in the heart, affecting diastolic function (Westermann et al., 2011). On the other hand, it could damage endothelial cell function, thus affecting the interstitial connectin-43 mediated cardiomyocyte coupling mechanism, resulting in reduced bioavailability of nitric oxide (NO), and thereby inhibiting the exogenous cyclic adenosine monophosphate (cGMP) signaling pathway (Vettel et al., 2014), promote endothelium-mesenchymal transformation process (Murdoch et al., 2014), eventually lead to the increase of fibroblast and myofibroblast content, induce ventricular remodeling, and gradually progress to HFpEF. Because ACEI/ARB (Cleland et al., 2006; Massie et al., 2008), β-blocker (Conraads et al., 2012), aldosterone receptor antagonist (Edelmann et al., 2013), ARNI (Solomon et al., 2019) and other drugs used for HFrEF have been proved to be ineffective for HFpEF, in recent years, scholars have begun to seek specific drugs based on the pathophysiological mechanism of HFpEF to achieve treatment goal. Clinical studies on the treatment of HFpEF by inhibiting inflammatory response (Glezeva and Baugh, 2014), increasing bioavailability of NO (Reddy et al., 2017), and inhibiting cGMP degradation (Liu et al., 2017) was carried out, but no breakthrough has been made. More new targeted drugs need to be further explored in the future. In the case that western drugs cannot achieve the expected efficacy of HFpEF through single-target intervention, the multi-target mechanism of Chinese medicine may have certain advantages. Modern pharmacology studies found that the drugs huangqi and danshen (the composition of Qishen Yiqi Dripping Pill) could down-regulate the expression of myocardial cell calcium transport or release protein CaMK II, PKA, NCX1 and RyR2, upregulate the expression of SERCA2a and PLB, thereby improve the function of myocardial cell calcium transients, maintaining myocardial cell calcium homeostasis, and then improve the myocardial diastolic function (Sun et al., 2012; Liu, 2017). The Qishen Yiqi Dripping Pill may reduce inflammation and oxidative stress in HFpEF patients, reduce myocardial stiffness, improve myocardial compliance, and inhibit myocardial cell apoptosis by regulating the IL-17 pathway, TNF pathway, MAPK pathway and GMP-PKG pathway (Zhang et al., 2020). Therefore, Qishen Yiqi Dripping Pill has great advantages and potential in treatment of HFpEF, but its mechanism of action is still not completely clear, which needs further research in the future.

This study is the first to systematically review the efficacy and safety of Qishen Yiqi Dripping Pill in the treatment of HFpEF. There have been three systematic reviews of TCM treatment for HFpEF in the past (Xu et al., 2015; Liu and Xu, 2018; Wang et al., 2018), but none of them strictly defined the treatment time of the included studies. The course of treatment in most of the studies was about 1 month, and some was only 2 weeks. However, HFpEF is a chronic disease, the short-term treatment is often difficult to achieve satisfactory curative effects. At the same time, the outcomes of the above three systematic reviews were relatively small, and among which two did not limit the types of Chinese herbs used in the intervention group (Liu and Xu, 2018; Wang et al., 2018), leading to a large difference in the intervention measures among the included studies, as well as a unreliable conclusions of the meta-analysis. Compared with the above three systematic reviews, our review selected more outcomes, the treatment duration of the included studies was longer and TCM therapies of the intervention group was strictly restricted to only Qishen Yiqi Dripping Pill. It is beneficial to improve the quality of included studies, reduce heterogeneity, and improve the reliability of the meta-analysis results. However, there are still following limitations: Firstly, the overall quality of the included studies is low, the description of random sequence generation procedure, allocation concealment procedure, blinding method and other aspects is not comprehensive, and all of them are small sample studies. These factors affect the credibility of the results to some extent. Secondly, placebos are often used as control treatment in clinical trials. The most common effect of placebo is regulating the autonomic nervous system by affecting patient’s psychological state, thus affecting body’s function. As we have mentioned above, the occurrence of HFpEF is closely related to autonomic nervous system dysfunction, so the placebo effect should be fully considered when evaluating the effectiveness of drug therapy for HFpEF. However, all the included studies in our review did not use placebo control, which made it impossible to fully verify the effectiveness of Qishen Yiqi Dripping Pill and damaged the reliability of our conclusion. Thirdly, the dose of Qishen Yiqi Dripping Pill used in the intervention group was completely consistent, so it is impossible to evaluate the dose-effect relationship of Qishen Yiqi Dripping Pill in the treatment of HFpEF. Fourthly, due to the small number of included studies, it is impossible to evaluate whether there is publication bias. Fifthly, no matter what intervention measures are taken, the ultimate goal is to improve prognosis. The studies included in our review lacks the evaluation of incidence of long-term adverse events, resulting in the influence of Qishen Yiqi Dripping Pill on the prognosis of HFpEF patients cannot be confirmed.

Based on the above discussion, we suggested that the TCM clinical study design in the future should strictly follow the CONSORT (Consolidated Standards of Reporting Trials). The researchers should pay more attention to the long-term prognosis evaluation. At the same time, we expect more large sample, multicentre, long-term randomized and double-blind controlled trials to provide more sufficient evidence for the treatment of HFpEF by Qishen Yiqi Dripping Pill.

## Conclusion

Current evidence suggests that Qishen Yiqi Dripping Pill may be effective in the treatment of HFpEF. However, due to the low quality of the included studies, lack of placebo control, large heterogeneity among different studies, and great possibility of publication bias, the results of our review should be evaluated with more prudence, more high-quality clinical studies are needed to verify the conclusion in the future. In addition, the safety of Qishen Yiqi Dripping Pill remains uncertain, further assessment is required in the future.

## Data Availability

The datasets presented in this study can be found in online repositories. The names of the repository/repositories and accession number(s) can be found in the article/Supplementary Material.
