# Supplementary material for: Efficacy and Safety of Qishen Yiqi Dripping Pill for Heart Failure With Preserved Ejection Fraction: A Systematic Review and Meta-Analysis
Source: Front Pharmacol. 2021 Feb 9;11:626375. doi: 10.3389/fphar.2020.626375 (PMC7900630; doi:10.3389/fphar.2020.626375)
Supplement: Supplementary file 1 [file datasheet1.doc]

| **SUMMARY TABLE OF THE STUDIES INCLUDED**. | | | | | |
| --- | --- | --- | --- | --- | --- |
| **Study** | **Formulation** | **Source** | **Species, concentration** | **Quality control reported**  **(Y/N)** | **Chemical analysis reported**  **(Y/N)** |
| He SL et al.  2015 | Huang qi, Dan shen, San qi, Jiang xiang | Tianjin Tianshili Pharmaceutical Co., Ltd. | - Astragalus propinquus Schischkin, concentration uncertainty - Salvia miltiorrhiza Bunge, concentration uncertainty - Panax pseudo-ginseng, concentration uncertainty - Dalbergia odorifera,   concentration uncertainty | Y – Prepared according to National Drug Standards of China Food and Drug Administration (YBZ04332003-2008Z) | N |
| Hou JW et al.  2019 | Huang qi, Dan shen, San qi, Jiang xiang | Tianjin Tianshili Pharmaceutical Co., Ltd. | - Astragalus propinquus Schischkin, concentration uncertainty - Salvia miltiorrhiza Bunge, concentration uncertainty - Panax pseudo-ginseng, concentration uncertainty - Dalbergia odorifera,   concentration uncertainty | Y – Prepared according to National Drug Standards of China Food and Drug Administration (YBZ04332003-2008Z) | N |
| Hu JH et al.  2015 | Huang qi, Dan shen, San qi, Jiang xiang | Tianjin Tianshili Pharmaceutical Co., Ltd. | - Astragalus propinquus Schischkin, concentration uncertainty - Salvia miltiorrhiza Bunge, concentration uncertainty - Panax pseudo-ginseng, concentration uncertainty - Dalbergia odorifera,   concentration uncertainty | Y – Prepared according to National Drug Standards of China Food and Drug Administration (YBZ04332003-2008Z) | N |
| Li X et al.  2014 | Huang qi, Dan shen, San qi, Jiang xiang | Tianjin Tianshili Pharmaceutical Co., Ltd. | - Astragalus propinquus Schischkin, concentration uncertainty - Salvia miltiorrhiza Bunge, concentration uncertainty - Panax pseudo-ginseng, concentration uncertainty - Dalbergia odorifera,   concentration uncertainty | Y – Prepared according to National Drug Standards of China Food and Drug Administration (YBZ04332003-2008Z) | N |
| Qiu YH et al.  2016 | Huang qi, Dan shen, San qi, Jiang xiang | Tianjin Tianshili Pharmaceutical Co., Ltd. | - Astragalus propinquus Schischkin, concentration uncertainty - Salvia miltiorrhiza Bunge, concentration uncertainty - Panax pseudo-ginseng, concentration uncertainty - Dalbergia odorifera,   concentration uncertainty | Y – Prepared according to National Drug Standards of China Food and Drug Administration (YBZ04332003-2008Z) | N |
| Song SY et al.  2020 | Huang qi, Dan shen, San qi, Jiang xiang | Tianjin Tianshili Pharmaceutical Co., Ltd. | - Astragalus propinquus Schischkin, concentration uncertainty - Salvia miltiorrhiza Bunge, concentration uncertainty - Panax pseudo-ginseng, concentration uncertainty - Dalbergia odorifera,   concentration uncertainty | Y – Prepared according to National Drug Standards of China Food and Drug Administration (YBZ04332003-2008Z) | \  N |
| Zhang JL et al. 2013 | Huang qi, Dan shen, San qi, Jiang xiang | Tianjin Tianshili Pharmaceutical Co., Ltd. | - Astragalus propinquus Schischkin, concentration uncertainty - Salvia miltiorrhiza Bunge, concentration uncertainty - Panax pseudo-ginseng, concentration uncertainty - Dalbergia odorifera,   concentration uncertainty | Y – Prepared according to National Drug Standards of China Food and Drug Administration (YBZ04332003-2008Z) | N |
| Zhang KX et al.  2019 | Huang qi, Dan shen, San qi, Jiang xiang | Tianjin Tianshili Pharmaceutical Co., Ltd. | - Astragalus propinquus Schischkin, concentration uncertainty - Salvia miltiorrhiza Bunge, concentration uncertainty - Panax pseudo-ginseng, concentration uncertainty - Dalbergia odorifera,   concentration uncertainty | Y – Prepared according to National Drug Standards of China Food and Drug Administration (YBZ04332003-2008Z) | N |
