# Supplementary figures and images for: Efficacy and Safety of Qishen Yiqi Dripping Pill for Heart Failure With Preserved Ejection Fraction: A Systematic Review and Meta-Analysis
Source: Front Pharmacol. 2021 Feb 9;11:626375. doi: 10.3389/fphar.2020.626375 (PMC7900630; doi:10.3389/fphar.2020.626375)

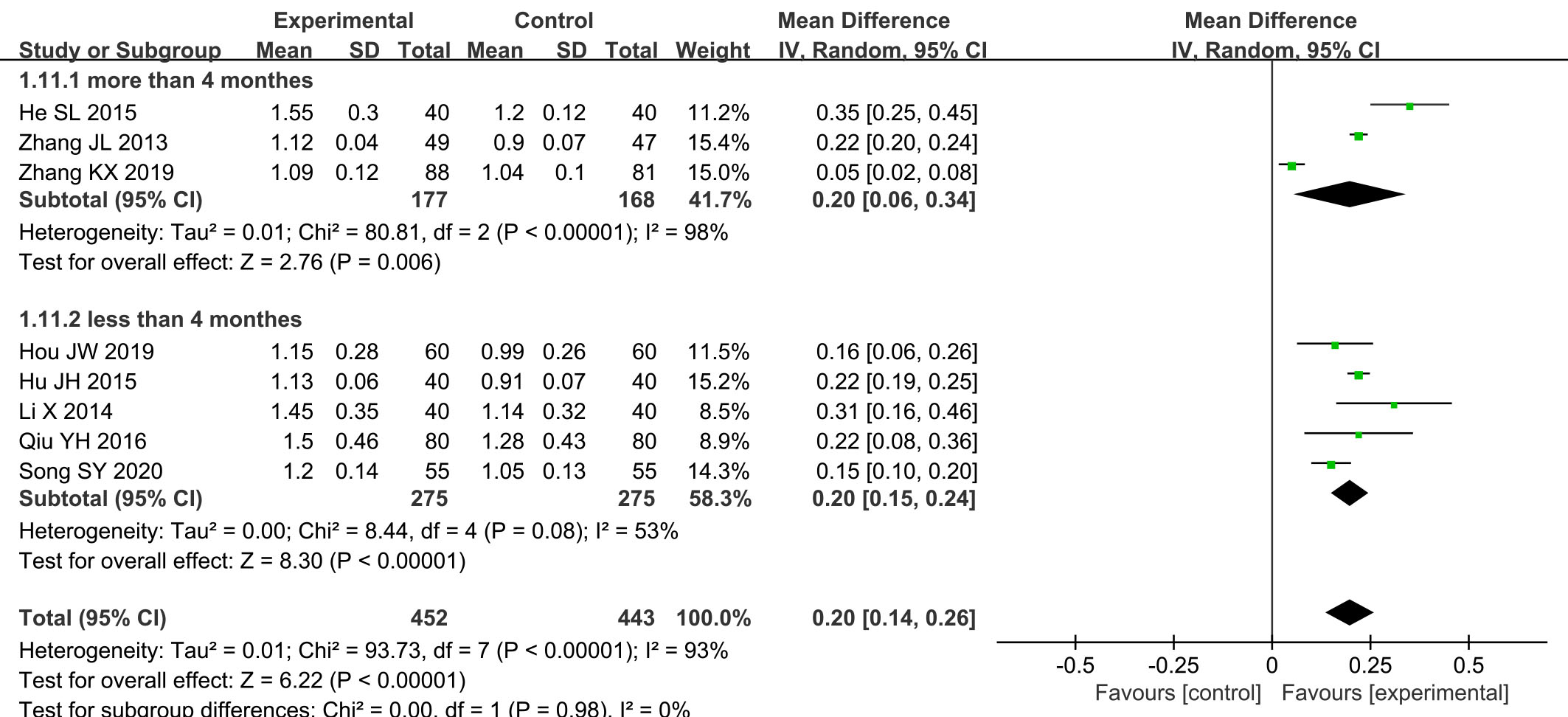

Supplement: Supplementary file 2 [file image1.jpeg]

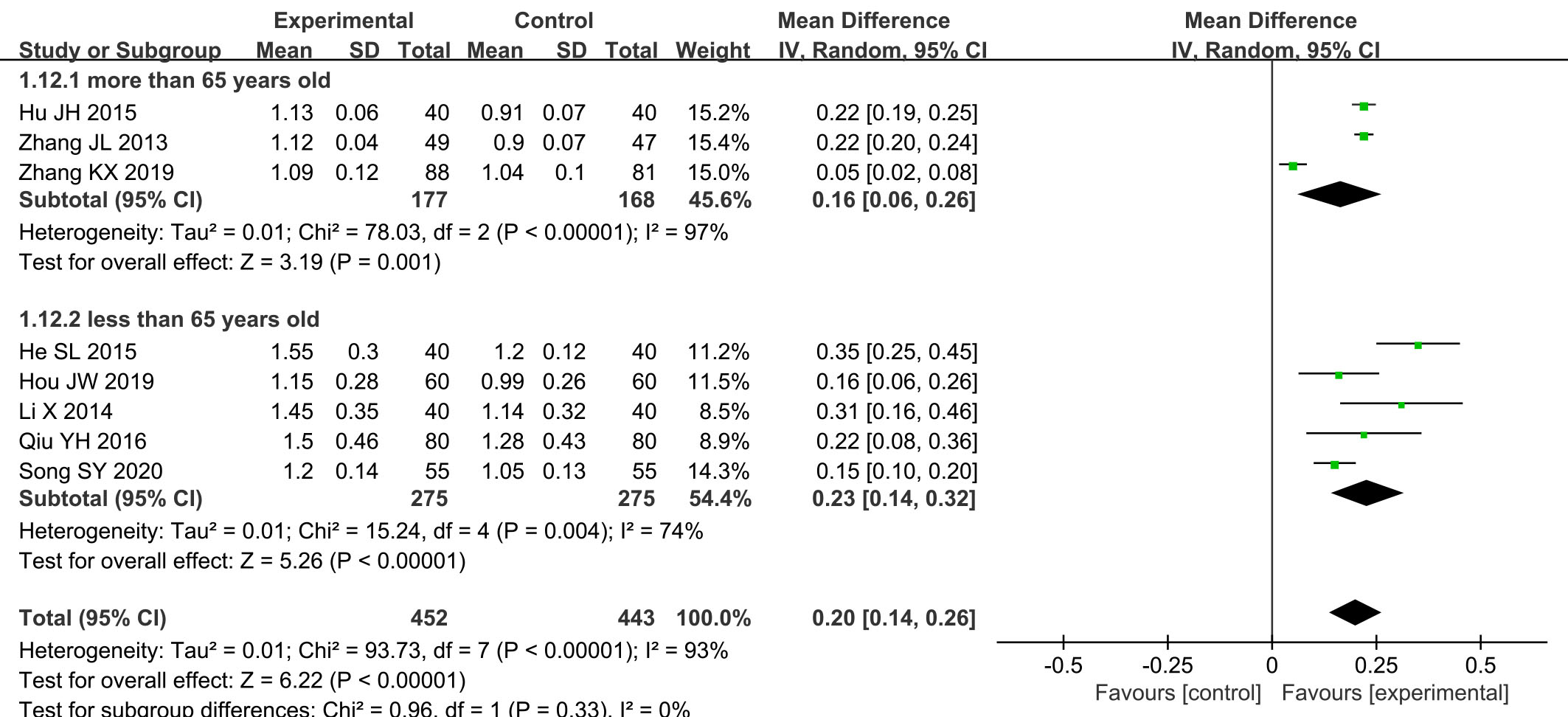

Supplement: Supplementary file 3 [file image2.jpeg]

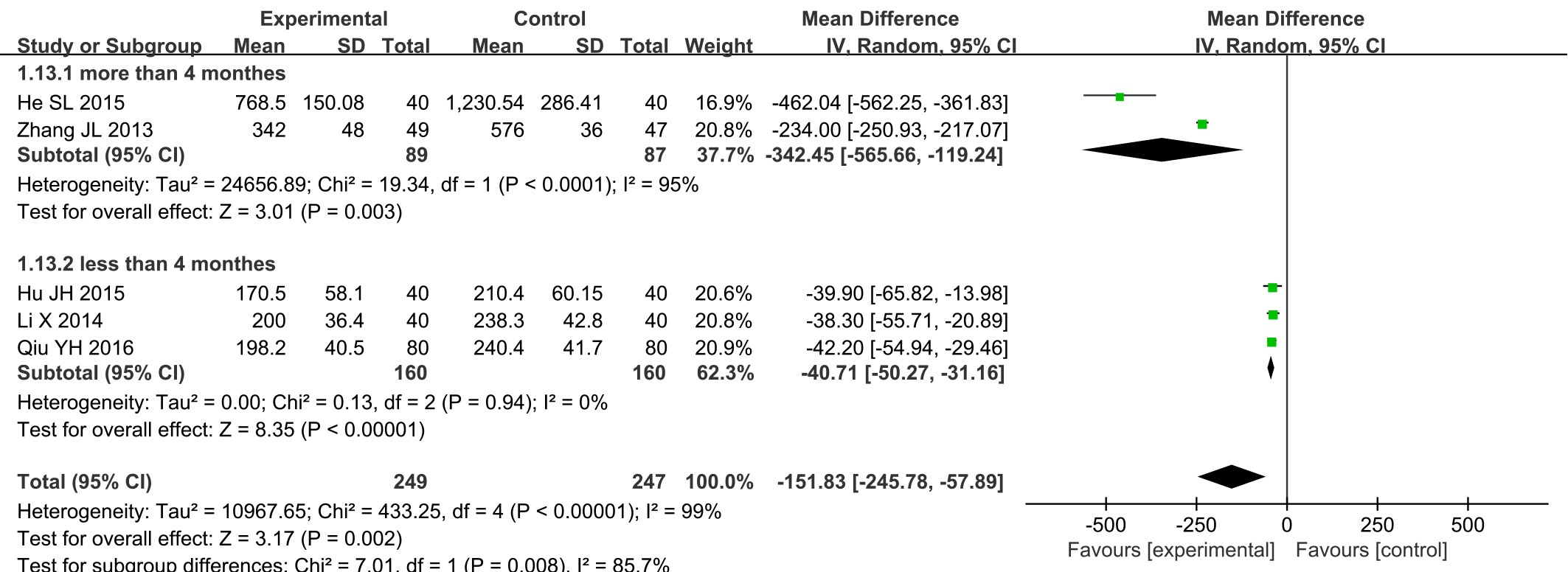

Supplement: Supplementary file 4 [file image3.jpeg]

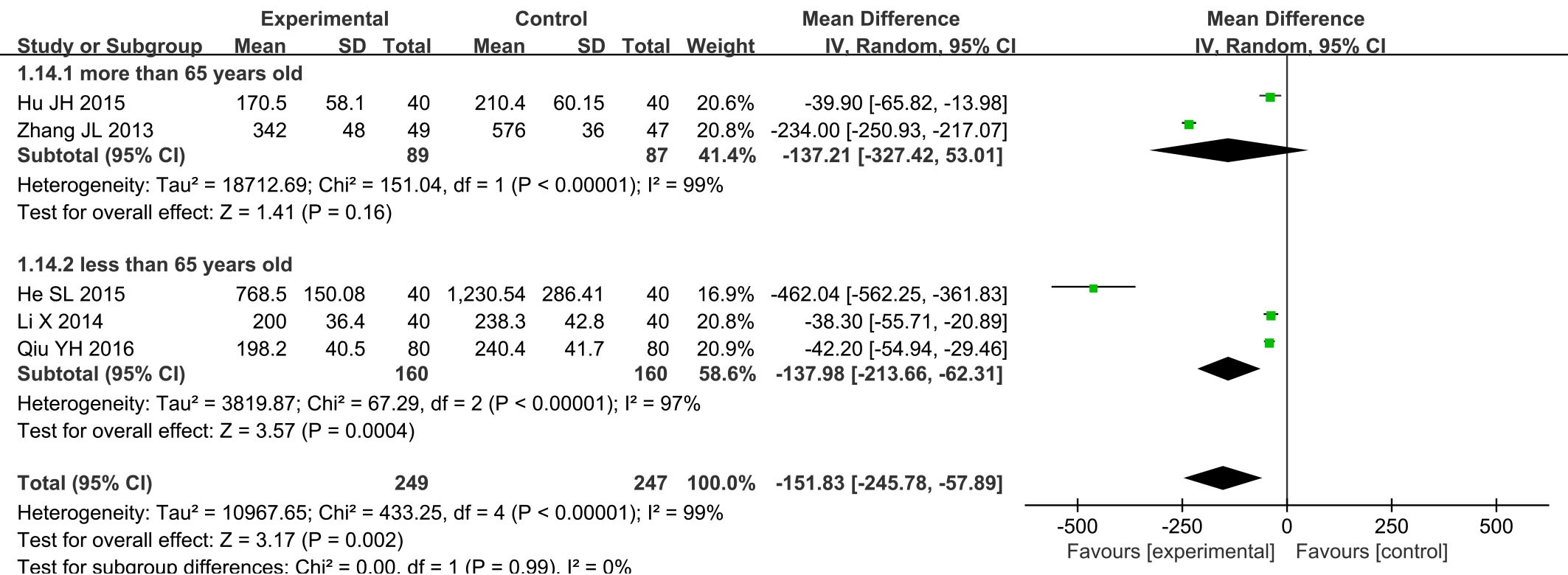

Supplement: Supplementary file 5 [file image4.jpeg]

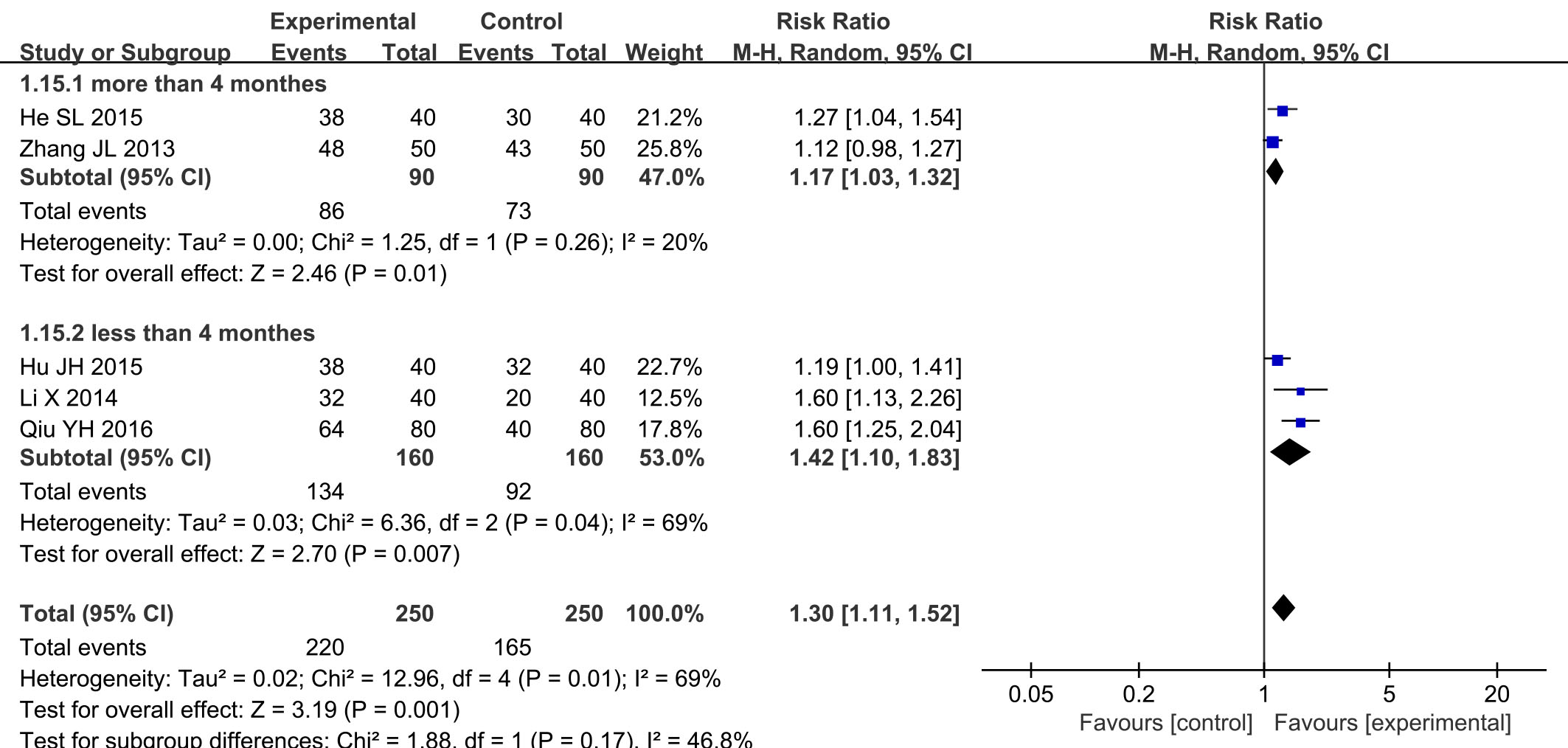

Supplement: Supplementary file 6 [file image5.jpeg]

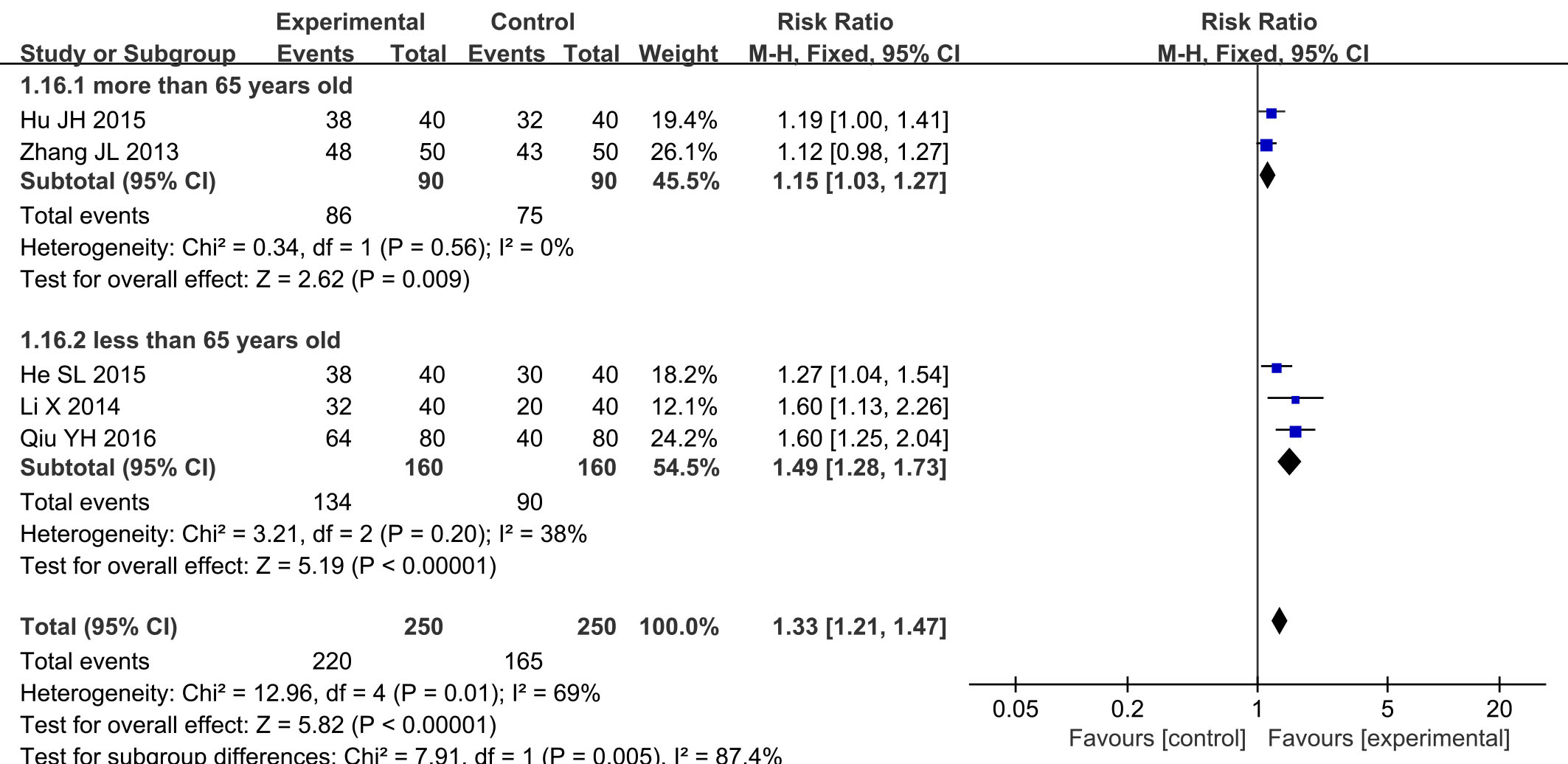

Supplement: Supplementary file 7 [file image6.jpeg]

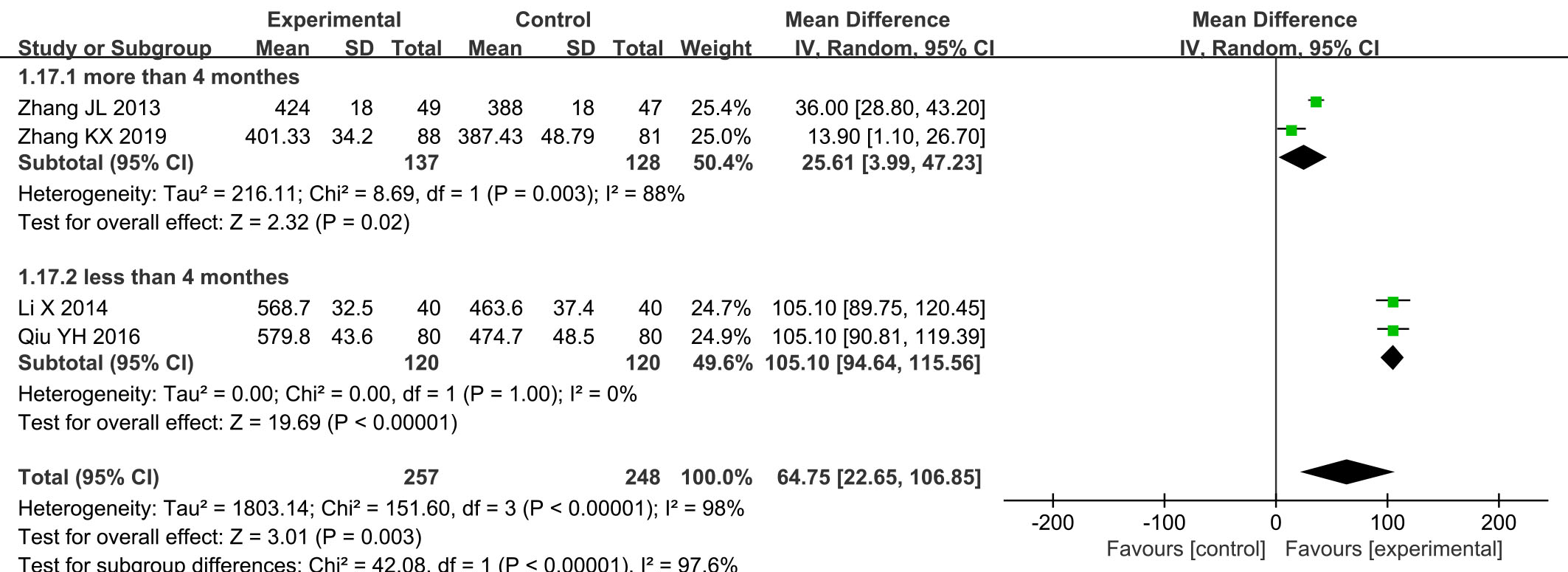

Supplement: Supplementary file 8 [file image7.jpeg]

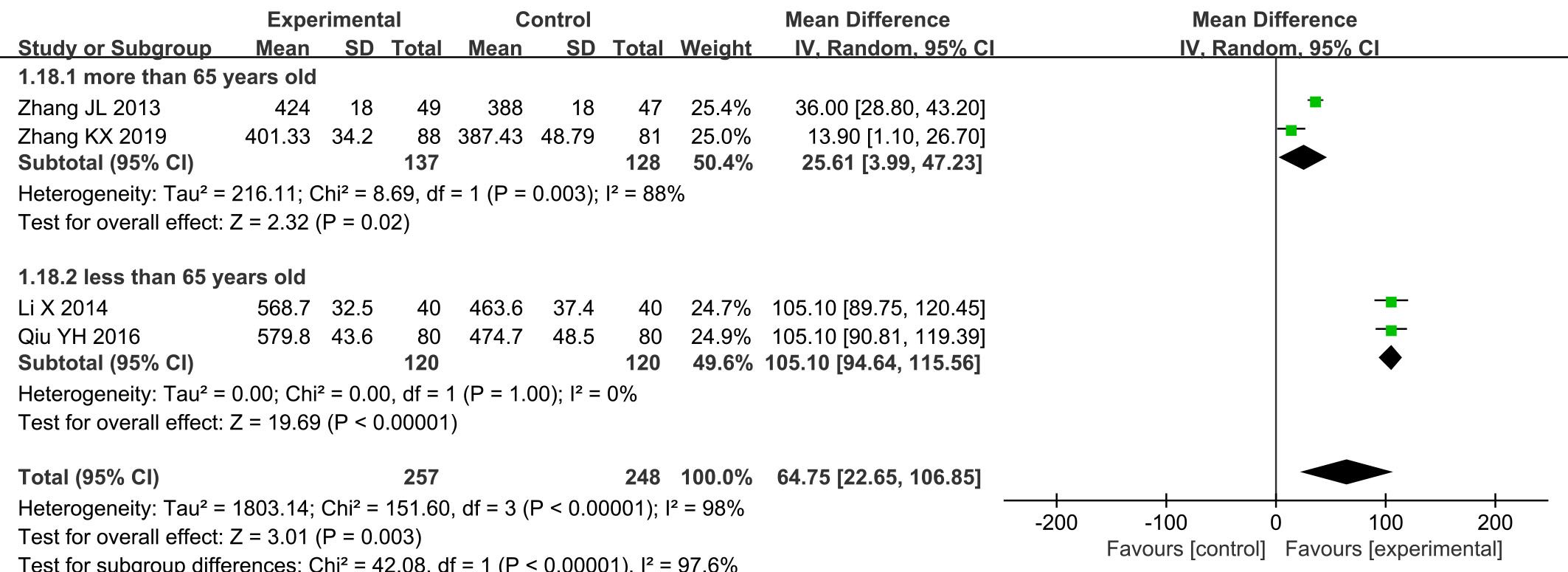

Supplement: Supplementary file 9 [file image8.jpeg]
